# A Case of Stone in the Bladder

**Published:** 1817-07

**Authors:** 


*For the London Medical and Physical Journal.*

### *A Case of Stone in the Bladder;* related by the PATIENT.

THE medicine I have used, for twenty-one months, was Adams's solvent for the stone and gravel, prepared by S. Perry, Esq. After trying it six months, and consulting him on the propriety of it, I added the use of Henry's calcined magnesia, occasionally, with the view of correcting the acidity with which the stomach was sometimes troubled. At the end of the first-mentioned time, I was somewhat easier, in general; but had discharged no part of the stone, the presence of which had been ascertained by sounding. About this time, I had several alarming discharges of blood at at the nose; and my medical assistant pronounced the crassamentum of the blood to have been reduced.

Now the use of the solvent was given up, but the magnesia was continued, but irregularly, for twenty-one months more; about which time, after a short ride, in much pain, I discharged, with some blood, a fragment of stone, convex and polished on one side, concave on the other,—in length 7-16 of an inch, in breadth 5-16, and in thickness 3-16 of an inch. Soon after this, I discharged another fragment; and, about a fortnight after, another, not much less in dimensions than the first. In the course of twelve months, about thirty were discharged, of different dimensions, from the size of the first to the smallest that could be ascertained to be a fragment, with a little mucus, containing some gritty substance, and sometimes an impalpable whitish powder. The discharges, after the first, were not accompanied by much pain, not even when a piece was detained in the urethra for three hours. And even now, in the present year, about eighteen months after the first discharge, I get rid of a small fragment occasionally, and sometimes in places where they cannot be searched for.

Most of the fragments appeared to me to be composed of different laminæ, and to be of the same substance as some pebbles discharged before taking the solvent, which a French surgeon, who pretended to analyze them, pronounced to be composed of uric acid and urat of ammonia. But some of them are of a darker hue, and not so firm; so that I have had, it should seem, at least two calculi broken. Indeed six months before the first fragment came away, it seemed, by the sensation and the gurgling of the urine, that the bladder contained more than one. The corners of many of the pieces were worn, it should seem, by rubbing against others.

Still I carry some more, I am certain, and I think some of no small size; and the nucleus of some of the fragments, the mark of which was clearly visible in them, still remains, or was not visibly discharged.

Those who know me will depend upon the truth of the above statements, and I believe there is no mistake in them. But I pretend not to say, that the solvent, which was taken regularly for twenty-one months, or that the magnesia, taken irregularly, and still continued with distilled water, was the cause of the separation of the calculi. But I took no other remedy. If the scientific physician or surgeon can shew the cause (and there must have some cause operated), I shall feel obliged by his observations and gratuitous advice.

All I can say is, that, since the discharge of many fragments, ments, I have been generally better, especially with small exercise, for which I am thankful to God.

*Moretonhampstead;*

*Feb.* 1, 1817.

P.S.—A few days after the above date, twelve fragments, of various sizes, were discharged in the course of twenty-four hours, and were found, by analyzing, to consist of uric acid.

